# Coordinated Development of Muscles and Tendon-Like Structures: Early Interactions in the *Drosophila* Leg

**DOI:** 10.3389/fphys.2016.00022

**Published:** 2016-02-04

**Authors:** Cedric Soler, Lilia Laddada, Krzysztof Jagla

**Affiliations:** Genetics, Reproduction and Development Laboratory (GReD) Genetics, Reproduction and Development Laboratory, Institut National de la Santé et de la Recherche Médicale U1103, Centre National de la Recherche Scientifique UMR6293, Clermont UniversityClermont-Ferrand, France

**Keywords:** tendon, muscle development, leg disc, tissue interactions, *Drosophila*

## Abstract

The formation of the musculoskeletal system is a remarkable example of tissue assembly. In both vertebrates and invertebrates, precise connectivity between muscles and skeleton (or exoskeleton) via tendons or equivalent structures is fundamental for movement and stability of the body. The molecular and cellular processes underpinning muscle formation are well-established and significant advances have been made in understanding tendon development. However, the mechanisms contributing to proper connection between these two tissues have received less attention. Observations of coordinated development of tendons and muscles suggest these tissues may interact during the different steps in their development. There is growing evidence that, depending on animal model and muscle type, these interactions can take place from progenitor induction to the final step of the formation of the musculoskeletal system. Here, we briefly review and compare the mechanisms behind muscle and tendon interaction throughout the development of vertebrates and *Drosophila* before going on to discuss our recent findings on the coordinated development of muscles and tendon-like structures in *Drosophila* leg. By altering apodeme formation (the functional *Drosophila* equivalent of tendons in vertebrates) during the early steps of leg development, we affect the spatial localization of subsequent myoblasts. These findings provide the first evidence of the developmental impact of early interactions between muscle and tendon-like precursors, and confirm the appendicular *Drosophila* muscle system as a valuable model for studying these processes.

In vertebrates, the progenitors of axial tendons arise from a dorsal subdomain of the sclerotome, called syndetome, that is immediately adjacent to the myotome from which myogenic cells originate (Brent et al., 2003). Crucial here is the fact that FGF signals emanating from the myotome are directly responsible for inducing the syndetome (Brent and Tabin, 2004; Brent et al., 2005; Chen and Galloway, 2014). Interactions between tendon and muscle progenitors thus take place in the very early steps of axial tendon development. On the other hand, the progenitors of limb and craniofacial tendons emerge independently of muscle progenitors (Kardon, 1998; Schweitzer et al., 2010). However, the muscles are subsequently required for further tendon development and maintenance (Edom-Vovard et al., 2002; Grenier et al., 2009). Thus, at either early or later stages of development, musculoskeletal formation in vertebrates is reliant on interactions between muscles and tendons.

The myogenesis process has been remarkably well preserved throughout the evolution. Various different models have been developed to study muscle development and muscle physiology, including insect models such as grasshopper and fly (Ho et al., 1983; Ball et al., 1985a; de Joussineau et al., 2012; Dobi et al., 2015). *Drosophila melanogaster* is the dominant genetic model used in studies of insect development, as transgenic flies are relatively easy to generate and there is a large range of genetic tools now available. Most of our knowledge of *Drosophila* muscle development stems from studies conducted on larval muscles (see Dobi et al., 2015 and de Joussineau et al., 2012 for review). Larval somatic muscles are set up during embryogenesis and have a very simplified pattern of 30 muscles repeated in each hemisegment. They originate from a subdivision of the mesoderm from which segregate three types of myoblasts, including the so-called Founder Cells (FC) and Fusion Competent Myoblasts (FCM) (Bate, 1990; Leptin, 1991; Baylies et al., 1998). Each FC fuses with several FCM to build a syncytial myotube (Rochlin et al., 2010; Abmayr and Pavlath, 2012; Kim et al., 2015). The expression of a distinct set of identity genes by the original FC determines the characteristic shape, size, position and innervation of each myofiber (Tixier et al., 2010; de Joussineau et al., 2012). Adult muscle precursors (AMP) arethe third type of myoblasts that are set aside during embryogenesis. AMPs are maintained quiescent and undifferentiated during embryogenesis and larval life, before proliferating then differentiating into adult muscles during metamorphosis (Bate et al., 1991; Broadie and Bate, 1991; Currie and Bate, 1991; Roy and VijayRaghavan, 1999). At both larval and adult stages, muscles are only effective when they are properly anchored to the exoskeleton through Muscle Attachment Sites (MAS). These tendon-like cells have been well investigated for larval muscles, yet only a few studies have focused on adult “tendogenesis” (Volk, 1999; Ghazi et al., 2000, 2003; Schnorrer and Dickson, 2004; Soler et al., 2004; Schweitzer et al., 2010).

## Development of the tendon-like structures in *Drosophila*

### Mechanisms of interaction between muscles and MAS during embryogenesis

Much as in vertebrates, *Drosophila* muscles have to be properly attached to the (exo-)skeleton in order to transmit the force generated by fiber contraction.

Invertebrate model organisms lack an internal skeleton, but somatic muscles interact with Epidermal Muscle Attachment (EMA) cells that are singled out from a cluster of exoskeleton cells called the apodeme (Ball et al., 1985b; Radnikow and Bässler, 1991; Volk, 1999). As muscles and apodemes connect through a secreted extracellular matrix, forming the equivalent of the Myo-Tendinous Junction (MTJ) in vertebrates, they are widely referred to as “tendons” in *Drosophila* studies (Volk, 1999; Schweitzer et al., 2010). Therefore, although invertebrate apodemes and vertebrate tendon cells do not share the same origin (ectodermal and mesodermal origin, respectively), they do ensure the same function.

As stated earlier, MAS formation has been well described in *Drosophila* for larval muscles (Volk, 1999; Schnorrer and Dickson, 2004; Schweitzer et al., 2010) in which studies report how the apodemes derive from specialized ectodermal cells. The initial differentiation of these cells is muscle-independent and requires ectodermal signals such as Wg, Hh, and EGF (Piepenburg et al., 2000; Hatini and DiNardo, 2001) to induce the expression of Stripe (Sr). Sr is the earliest known marker of tendon-like precursors. It encodes a transcription factor with a zinc-finger domain and shares sequence homologies with members of the vertebrate Egr family (Volk and VijayRaghavan, 1994; Frommer et al., 1996). Interestingly, EGR1 and EGR2 are involved in tendon-cell differentiation in vertebrate limbs (Lejard et al., 2011). In *Drosophila*, Sr is both necessary and sufficient for MAS induction by activating the expression of most of the MAS-specific genes (Becker et al., 1997; Vorbrüggen and Jäckle, 1997). The *Sr* gene encodes for two isoforms: SrA and SrB (Frommer et al., 1996). A low level of SrB isoform is required for induction of tendon-like precursors during early embryonic development (Becker et al., 1997; Vorbrüggen and Jäckle, 1997). These precursors then secrete signaling molecules that are involved in muscle guidance and attachment. For example, under Sr regulation, Slit ligand is expressed by tendon-like precursors and interacts with its receptors Robo 1 and 2 that are located on the membrane of specific muscles migrating toward their attachment sites (Kramer et al., 2001; Volohonsky et al., 2007). In turn, muscle fibers produce Vein, a neuregulin-like ligand that activates the EGFR pathway in tendon-like cells (Yarnitzky et al., 1997). EGFR pathway activation leads to an increase in SrB expression, which subsequently promotes splicing of the SrA isoform and leads to the terminal differentiation of the MAS and the establishment of the MTJ (Nabel-Rosen et al., 2002; Volohonsky et al., 2007). The tendon-like precursors that do not receive EGF signal from muscles lose MAS marker expression and eventually dedifferentiate. Thus, in a similar way to the craniofacial and limb tendons described in vertebrate studies, tendon-like precursors in *Drosophila* embryo are specified independently of muscle cells whereas the terminal phase of differentiation is muscle-dependent (Volk, 1999; Schnorrer and Dickson, 2004; Schweitzer et al., 2010).

### Adult muscles anchoring to cuticle within the thorax allows fly locomotion

The MAS development during the adult myogenesis in *Drosophila* has paid much less attention than in embryo. In adults, the main thoracic muscles are the flight muscles [including Direct Flight Muscles (DFM) and large Indirect Flight Muscles (IFM)] and the leg muscles (Miller, 1950; Fernandes et al., 1991; Soler et al., 2004) developing from AMP that are associated with wing and leg discs, respectively. Theses myoblasts are characterized by the expression of Twist that persists until metamorphosis (Bate et al., 1991; Broadie and Bate, 1991). The MAS of flight muscles develop from distinct groups of Sr-expressing cells from the wing disc epithelium (Fernandes et al., 1996). The pattern of flight muscle MAS is defined by the integration of several molecular signals including Notch, Wnt, and Dpp, and is regulated by the transcription factors Apterous and Achaete scute (Ghazi et al., 2000, 2003; Usui et al., 2004). In the leg, apodemes adopt their own particular shape. Sr-expressing epithelial cells invaginate inside the developing leg to form long internal structures (Soler et al., 2004). Remarkably, Twist-expressing myoblasts accumulate around the apodeme precursors long before they form syncytial fibers. This observation strongly suggests that muscle and tendon-like precursors interact with each other at an early stage in the developing leg (Soler et al., 2004). Similar long internal apodemes have been described in appendages of other insects (Ball et al., 1985b; Radnikow and Bässler, 1991) and more generally in several groups of arthropods, including crustaceans (Medler and Mykles, 2015). Despite their value as models for histological and physiological studies, the lack of genetic tools for these organisms precludes any attempt at systematic molecular or genetic analysis.

## Perspectives

### *Drosophila* appendicular myogenesis as a model for early interactions between muscle and muscle attachment site progenitors

**Figure 1:**
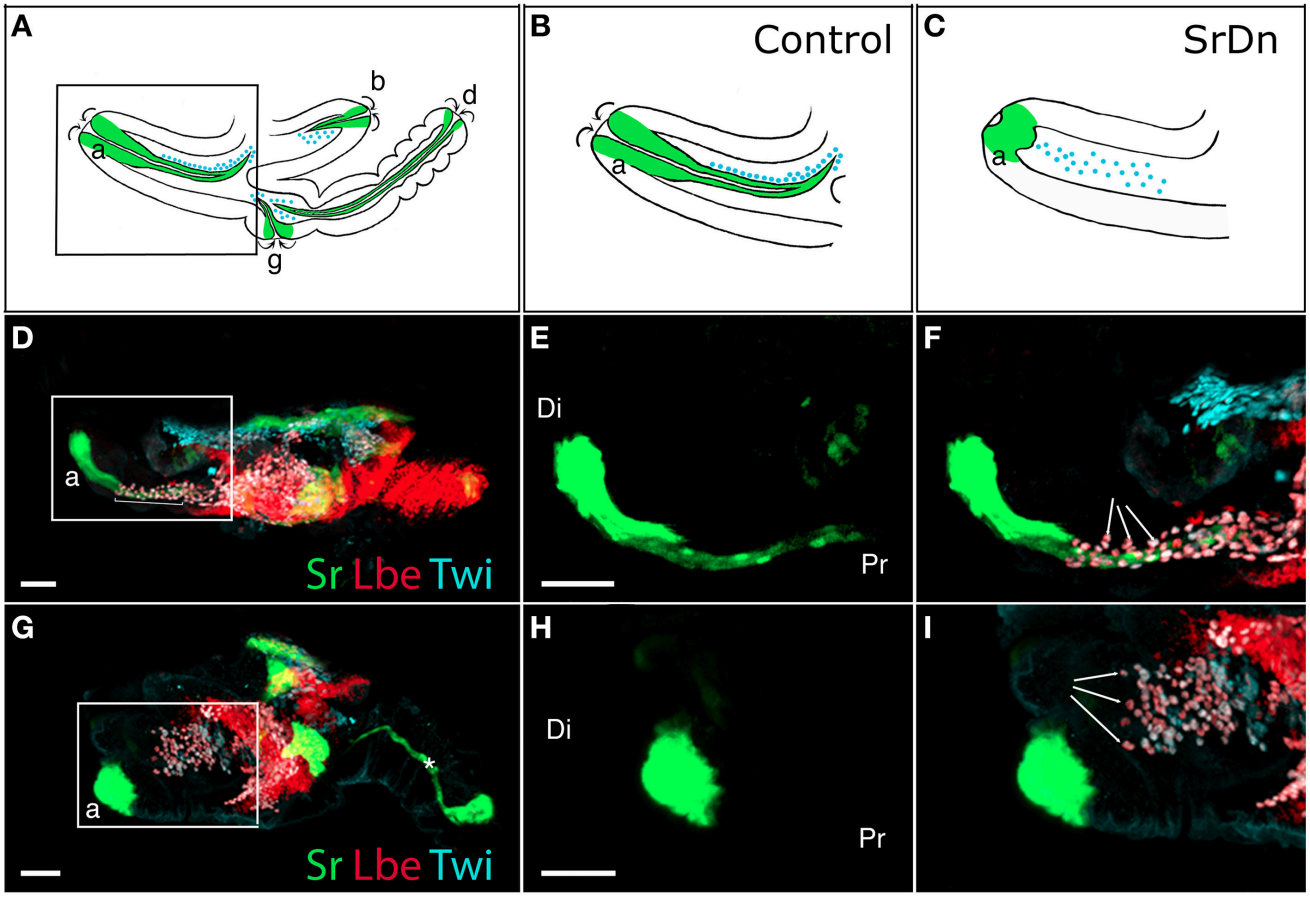
**Disrupting apodeme development affects myoblast spatial organization. (A–C)** Drawings of sagittal views at 5 h After Pupae Formation (APF) of a whole wild-type leg disc **(A)** with a focus on the tibia levator tendon/apodeme (a) of the dorsal femur segment **(B)** and a leg disc for which apodeme invagination in the dorsal femur was affected **(C)**. Only some apodemes (green) and their associated myoblasts (blue) are represented. Note that their invagination (curved arrows) goes on to form a lumen. **(D–I)** Leg discs at 5 h APF from Tub-Gal80^TS^;Stripe-gal4>UASGFP **(D–F)** and Tub-Gal80^TS^;Stripe-gal4>UASGFP, UAS-Stripe^DN^ **(G–I)**. Apodemes are visualized by GFP and myoblasts stained for Twist (in cyan) and Lbe (in red). At this stage, the leg disc elongates along the proximo-distal axis (Pr-Di). **(D)** Control leg disc showing tibia levator tendon (a) invaginating from distal to proximal ends of the dorsal femur (insert), the myoblasts are aligned along this apodeme (brackets). **(E,F)** Enlargements of the box region in **(D)** showing apodeme developing in the femur **(E)** and the myoblasts organized around it (arrows in **F**). **(G)** When Stripe^DN^ is expressed in apodemes, they fail to develop correctly, with the result that the apodeme (a) in the dorsal femur is unable to invaginate to form a long internal structure (insert). **(H,I)** Enlargements of the box region in **(G)** showing aborted apodeme in femur **(H)**. Myoblasts in this region do not appear to align in the proximo-distal axis and seem to distribute in random directions into the femur segment (arrows in **I**). Note that in **(G)**, the main apodeme in the tarsus (star) does invaginate despite expressing UAS-Stripe^DN^ at 5h APF. This first apodeme invaginates as early as L3, at which stage we shifted the larvae from 18 to 29°C to allow Gal4 expression, which thus makes it very likely that it undergoes invagination before Stripe^DN^ protein accumulation could have any effect. Myoblasts associated with this apodeme are not in focus. Scale bar = 30 μm. Apodeme and muscle annotations: (a) tibia levator tendon in dorsal femur (associated muscle: tibia levator muscle), (b) tibia depressor tendon in ventral femur (associated muscle: tibia depressor muscle), (d) long tendon in tarsus (associated muscles: long tendon muscle 1, tarsus reductor muscles 1 and 2), (g) tarsus levator tendon in dorsal tibia (associated muscle: tarsus levator muscle). See Soler et al. (2004) for more detailed annotations.

The leg muscle system of *Drosophila* is a complex structure that counts 14 distinct muscles (Miller, 1950; Soler et al., 2004). Unlike larval muscles, each leg muscle is composed of several fibers organized around a specific long internal apodeme and attached from one side to this tendon-like structure and from the other side to the cuticle (via embryonic-like apodemes) (Soler et al., 2004). This particular pattern of multifiber muscles enables precise and coordinated movements of all nine articulated segments of the leg. In addition to being functionally comparable to vertebrate limb muscles, there are other morphological parallels to draw with the musculoskeletal system of the vertebrate limb. For example, in vertebrates, long tendons of the limb extend from the most distal region (paw) to more proximal segments (arm/leg) where they are associated with their corresponding muscles, allowing articulation of the distal limb part (Kardon, 1998; Huang et al., 2015). Similar schemes have been developed in insect legs, with some long internal apodemes running through several leg segments (Ball et al., 1985b; Radnikow and Bässler, 1991; Soler et al., 2004). In *Drosophila*, the leg muscles derive from adepithelial cells that are located at the surface of leg imaginal discs while the tendon-like precursors are groups of Stripe-positive cells belonging to the disc epithelium. Both tendon-like and muscle precursors are set aside before metamorphosis and leg development. More strikingly, a specialized subpopulation of muscle precursors identified as Founder Cells (FCs) are specified as early as the third larval instar (L3) near these tendon-like precursors (Soler et al., 2004; Maqbool et al., 2006). We have previously reported (Soler et al., 2004) that at the early pupa stage when a disc evaginates to form a 3D leg structure, the tendon-like precursors invaginate inside the developing leg and specific FC myoblasts follow this pattern (Figures 1A,B). This observation suggested that invaginating tendon-like precursors of the leg could interact with FCs to accurately localize them, and could thus play a crucial role during the early steps of leg muscle development. This hypothesis is further supported by our recent data indicating that tendon-like precursors are indeed required for proper patterning of appendicular myoblasts (Figure 1).

### How could disruption of tendon-like precursors affect muscle FCs in the developing leg?

**Figure 2:**
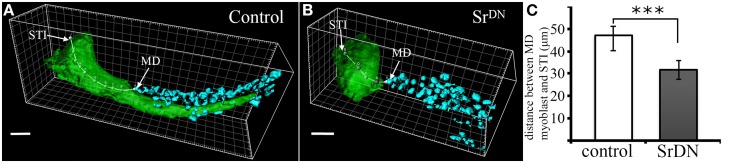
**Spatial distribution of myoblasts after Sr^DN^ expression. (A–B)** Confocal 3D rendering of tibia levator tendon (in green) and associated myoblasts (in cyan) in the dorsal femur of a leg disc at 5 h APF. **(A)** shows invaginating apodeme for the control sample with the spatial distribution of myoblasts to be compared against myoblast distribution after affecting apodeme development **(B)**. Distance from the Most Distal myoblast (MD) to the Site of Tendon Invagination (STI) was measured using Imaris MeasurementPro through the 3D volume of the apodeme. Scale bar = 10 μm. **(C)** Quantification of MD-to-STI distance. Mean distance is significantly reduced in Sr^DN^ samples (31, 67 μm; *n* = 11) compared to control samples (46, 95 μm; *n* = 8). Error bars represent standard deviation, ^***^ *p*-value < 0.001 using the Student's *t*-test.

Our earlier work (Maqbool et al., 2006) showed that *ladybird early* (*lbe*), an ortholog of *Lbx1*, which is a key regulator of appendicular myogenesis in vertebrates (Buckingham et al., 2003), is expressed in different sub-populations of myoblasts characterized by Twist expression (Bate et al., 1991; Broadie and Bate, 1991). From the third instar larval stage, the spatial distribution of these myoblasts revealed a highly stereotyped pattern that underpins the formation of defined muscles in the adult leg. We also showed that *lbe* and its paralog *ladybird late (lbl)* genes are required for proper patterning of leg muscles and that different levels of Lbe protein contribute to myoblast diversity within the leg (Maqbool et al., 2006). As these myoblasts also express the Dumbfounded-lacZ reporter gene, they are very likely the equivalent of embryonic FC in embryo (Soler et al., 2004; Maqbool et al., 2006). More strikingly, in each segment, Lbe-positive groups of myoblasts lie close to Sr-positive tendon-like precursors. This distribution is particularly obvious in the dorsal femur where Lbe and Twist-expressing myoblasts accumulate next to the tibia levator tendon (tilt). At the beginning of metamorphosis, these myoblasts remain associated with the tilt as it begins to invaginate. Five hours After Pupae Formation (APF), they progressively align all along this internal apodeme. Figures 1A,B illustrates this spatial distribution of myoblasts and their association with invaginating apodemes. In dissected leg discs, apodemes are visualized by GFP expression driven by Sr-Gal4 driver and myoblasts by immunostaining against Lbe and Twist (Figures 1D–F). In order to determine whether invaginating apodeme could influence myoblast behavior, we challenged apodeme development by over-expressing a dominant-negative form of Sr (Sr^DN^) (Vorbrüggen and Jäckle, 1997) using the Sr-Gal4 driver. As Sr is also involved in MAS development in embryos, we used a ubiquitous temperature-sensitive Gal80^ts^ allele to repress Sr^DN^ expression until mid-L3 stage. Figures 1G–I shows that Sr^DN^ expression affects apodeme formation at 5 h APF. In particular, compared to controls (Figure 1E) tendon-like cells appear unable to form a long internal structure in the dorsal femur (Figure 1H). Even after disrupting apodeme development, Lbe expression was still detected in associated myoblasts, indicating that the occurrence of invaginating apodeme is not required to maintain the expression of this muscle identity gene (compare panels F and I in Figure 1). However, these myoblasts appeared disorganized within the everting segment when the apodeme is affected (Figure 1I) yet well aligned on the developing apodeme of the control leg disc (Figure 1F). This observation indicates that in the absence of invaginating tendon-like precursors, myoblasts are no longer correctly distributed within the observed segment, even though they still follow the segmental subdivision of the leg disc. To compare the spatial distribution of dorsal femur myoblasts between control leg discs and Sr^DN^ leg discs in which tilt apodeme is significantly affected, 3D reconstructions of several early pupa discs (5 h APF) were built and visualized using Imaris™ Software (Figures 2A,B). These 3D reconstructions were then used to measure the distance between the Most Distal myoblast (MD myoblast) and the Site of Tendon-like Invagination (STI) at the epithelium surface (distal femur) (Figure 2). This MD myoblast-to-STI distance was the parameter that showed least variation across Sr-DN samples. Our data show that, mean MD myoblast-to-STI distance is significantly higher in control leg discs (46,9 μm, *n* = 8) than after Sr^DN^ expression in tendon-like precursors (shortened to 31,67 μm, *n* = 11; Figure 2C). This result demonstrates that apodeme alteration leads to aberrant myoblast positioning in the leg discs of early pupa and may therefore impact the morphological and functional properties of the corresponding adult leg muscles. It remains to be elucidated whether muscle precursor distribution is controlled by direct interactions with the developing apodemes or whether their positioning is guided by physical constraints during leg disc evagination. It is reasonable to assume that in absence of apodeme, myoblasts would have a wider area in which to spread within the segment cavity. In both hypotheses, the data reported here show that in the early steps of leg development, apodemes are directly or indirectly required for proper patterning/organization of myoblasts that have previously been identified as FC (Soler et al., 2004; Maqbool et al., 2006).

## Discussion

Taken together, the data reported here show that in developing *Drosophila* legs, the invaginating tendon-like precursors orchestrate the spatial positioning of tightly associated appendicular founder myoblasts. Such early interactions between apodeme and muscle precursors have never before been observed during embryonic or flight muscle development. This study demonstrates that appendicular myogenesis is an attractive model for studying early interactions between tendon-like and muscle progenitors. In vertebrate limbs, muscle, and tendon induction occur independently, but the specification of tendon progenitors of the axial musculoskeletal system is directly dependent on the FGF ligand emanating from the adjacent myotome (Brent et al., 2003; Brent and Tabin, 2004). Thus, at least for certain muscles, early muscle/tendon interactions are required in both *Drosophila* and vertebrates and similar mechanisms may control certain aspects of these interactions. Note too that long internal apodemes have already been described in appendages of many invertebrates such as crustaceans (Medler and Mykles, 2015) and insects undergoing hemimetabolous development (incomplete metamorphosis with no pupal stage), as is the case of grasshoppers for which leg muscle system development around a long internal apodeme has been well described (Ball and Goodman, 1985; Ball et al., 1985b). In this model, muscle pioneers (equivalent of FC cells) are associated with ectodermal sites where the invagination begins (Ball et al., 1985b). However, the lack of specific markers precludes any attempt to determine whether these sites were already specified as tendon-like precursors and whether physical contacts were made at this stage. Using our *Drosophila* leg model, we showed that the presumptive leg muscle founders segregate close to the Sr-expressing apodeme, long before they start invaginating (Soler et al., 2004). Moreover, our most recent observations (CS, unpublished data) indicate that cell–cell contact indeed occurs as early as the third larval stage through cytoplasmic projections. The role of these connections has yet to be elucidated, but one possibility is that they are required to promote the segregation of FC and their identity. This hypothesis could be tested by abolishing the specification of the tendon-like precursors.

## Author contributions

CS, conception and design of the work, analysis and interpretation of data, drafting the work, final approval, and agreement for all aspects of the work; LL, acquisition, analysis, and interpretation of data, revising the work, final approval, and agreement for all aspects of the work; KJ, interpretation of data, revising the work, final approval, and agreement for all aspects of the work.

## Funding

This work was supported by the Institut National de la Santé et de la Recherche Médicale (INSERM), the Association Française contre les Myopathies (AFM), the Fondation pour la Recherche Medicale (FRM) and the national grant IDE-CELL-SPE from the Agence Nationale de la Recherche (ANR).

### Conflict of interest statement

The authors declare that the research was conducted in the absence of any commercial or financial relationships that could be construed as a potential conflict of interest.
